# A Novel Use of the “3-Day Rule”: Post-discharge Methadone Dosing in the Emergency Department

**DOI:** 10.5811/westjem.18030

**Published:** 2024-06-11

**Authors:** Jenna K. Nikolaides, Tran H. Tran, Elisabeth Ramsey, Sophia Salib, Henry Swoboda

**Affiliations:** * Rush University Medical Center, Substance Use Intervention Team, Department of Psychiatry and Behavioral Sciences, Chicago, Illinois; † Rush University Medical Center, Department of Emergency Medicine, Chicago, Illinois; ‡ Chicago College of Pharmacy, Midwestern University, Downers Grove, Illinois; § Queen’s University, Department of Emergency Medicine and Addictions Medicine, Kingston, Canada

## Abstract

**Introduction:**

Methadone is a medically necessary and lifesaving medication for many patients with opioid use disorder. To adequately address these patients’ needs, methadone should be offered in the hospital, but barriers exist that limit its continuation upon discharge. The code of federal regulations allows for methadone dosing as an inpatient as well as outpatient dispensing for up to three days to facilitate linkage to treatment. As a quality initiative, we created a new workflow for discharging patients on methadone to return to the emergency department (ED) for uninterrupted dosing.

**Methods:**

Our addiction medicine team changed hospital methadone policy to better allow hospitalization as a window of opportunity to start methadone. This necessitated the creation of a warm-handoff process to link patients to methadone clinics if that linkage could not happen immediately on discharge. Thus, our team created the “ED Bridge” process, which uses the “3-day rule” to dispense methadone from the ED post hospital discharge. We then followed every patient we directed through this workflow as an observational cohort for outcomes and trends.

**Results:**

Of the patients for whom ED bridge dosing was planned, 40.4% completed all bridge dosing and an additional 17.3% received at least one but not all bridge doses. Established methadone patients made up 38.1% of successful linkages, and 61.9% were patients who were newly started on methadone in the hospital.

**Conclusion:**

Improving methadone as a treatment option remains an ongoing issue for policymakers and advocates. Our ED bridge workflow allows us to expand access and continuation of methadone now using existing laws and regulations, and to better use hospitals as a point of entry into methadone treatment.

Population Health Research Capsule What do we already know about this issue?

*Federal regulations allow EDs to dispense methadone for opioid use disorder, and hospitals can use the 3-day rule to assist with linkages to methadone maintenance programs.*

What was the research question?

*We looked at the feasibility of using the ED as a post-acute care landing site to bridge patients’ methadone treatment in discharging hospitalized patients.*

What was the major finding of the study?

*Forty percent of patients (21/54) completed all bridge dosing, of whom 62% were newly initiated on methadone in the hospital.*

How does this improve population health?

*This workflow is a novel use of the 3-day rule to expand access to methadone via the ED.*

## INTRODUCTION

There are many regulatory barriers to initiating medications for opioid use disorder (MOUD) in traditional healthcare settings. Since treatment with methadone, an opioid agonist, or with buprenorphine, a partial opioid agonist, remains the standard of care for patients with opioid use disorder (OUD), there has been much focus recently on easing or circumnavigating barriers to facilitate linkage to treatment. While the passage of the 2023 Consolidated Appropriations Act removed the X-waiver requirement for buprenorphine prescribing,^1^ methadone dispensing remains restricted to opioid treatment programs (OTP). Given these restrictions on prescribing and other legal considerations, many hospitals are often hesitant to start and titrate methadone for inpatients with OUD.

Every year drug-related deaths continue to increase, and in 2021 over 80,000 people died of an opioid overdose.^2^ Underuse of MOUD is common among patients seen in the hospital despite evidence supporting emergency department (ED) and inpatient initiation as beneficial opportunities to start treatment.^3^^,^^4^ To address this deficit, our tertiary medical center created the Substance Use Intervention Team (SUIT) in 2018.^5^ The SUIT is comprised of emergency physicians who are dual- or triple-boarded in medical toxicology and/or addiction medicine, psychiatric nurse practitioners, social workers, a recovery support specialist, and a pharmacist; SUIT is available during business hours, Monday through Friday. The team is a comprehensive addiction medicine consult service, working toward increasing the recognition, treatment, and linkage to outpatient care for all substance use disorders. The SUIT offers all forms of MOUD, including buprenorphine and methadone. For patients who requested or preferred methadone, the dose titration was guided by the 2019 version of the California [CA] Bridge in-hospital methadone start protocol,^6^ tailored to each patient, with the most aggressive possible titration being 40 milligrams (mg) on day 1, 50 mg on day 2, and 60 mg on day 3, at which point, the dose was not increased until every five days.

Starting more patients on methadone necessitated the crafting of new policies and procedures at our center that would allow a warm handoff to methadone OTPs. The Code of Federal Regulations Title 21 restricts the dispensing of methadone to OTPs and specifies that methadone may be administered for three days in a healthcare setting for the purpose of alleviating withdrawal while arrangements are made to refer to treatment.^6^ It does not limit treatment to three days; however, if the patient is in the hospital for reasons other than withdrawal, MOUD can be used “to maintain or detoxify a person as an incidental adjunct to medical or surgical treatment of conditions other than addiction.”^7^ Therefore, methadone, if started while an inpatient, can be continued for the entirety of the stay. Prior to SUIT’s creation, our tertiary medical hospital had an internal policy that if methadone was started for a patient not previously enrolled in an OTP, the patient had to be weaned prior to discharge because of the prescribing limitation. Because weaning without further maintenance treatment only addresses the physical dependence in the short term while neglecting the chronic disease of OUD, it increases risk of relapse, fatal overdose, and all-cause mortality.^8^^–^^11^ This policy, although compliant with the law, was not evidence-based best practice.

The SUIT created a new policy and workflow that allowed the start of an inpatient titration of methadone for patients not previously enrolled in an OTP, arranged linkage to OTPs while still inpatient, and avoided weaning prior to discharge; if patients could not immediately be treated at an OTP upon discharge (due to gaps in treatment, including weekend or holiday closures), the ED is used as a post-discharge setting for continued dosing under the three-day rule to complete a warm handoff. This workflow was reviewed by our hospital’s pharmacy, compliance, and legal departments, all of which agreed that it complied with existing laws and helped us enact the change in hospital policy. Once this process was built, our team realized that it was also helpful for those patients in established OTPs who were discharged on weekends or holidays and couldn’t return to their OTP for dosing until the next business day.

Having the ED as a post-acute care landing site for methadone continuation helped avoid disruption of established MOUD as well as newly initiated MOUD. Because the new-start methadone titration was more aggressive than a typical outpatient initiation of methadone, when patients returned to the ED, the dose administered was their discharge dose and was not titrated in the ED to keep them at steady-state and to avoid a need for observation in the ED after dosing. During the timeframe this workflow was built and used, the OTPs in our city independently underwent changes. One OTP in particular agreed to honor hospital titrations on day 1 in their clinic if the patient brought discharge paperwork with them. The program became a preferred option for this workflow, although many patients either already used or requested other OTPs.

This article serves as a proof of concept and an observational cohort of all patients that SUIT directed to return to the ED for methadone dosing.

## METHODS

The setting of this study was our tertiary urban medical center. Patients identified as being in need of an “ED bridge” were included in this study if they were seen by the SUIT consult service; if they were identified as either already in a methadone OTP or newly started on methadone during the hospitalization and in need of enrollment in an OTP; and if the primary team determined that they would be discharged on a day where the patient would not immediately be able to get outpatient methadone dosing but with a plan in place for linking to an OTP within 72 hours of discharge. This identification usually happened on a Thursday or Friday in anticipation of a weekend discharge or for new methadone starts when an OTP appointment could not be made for the day after discharge. Social workers on the SUIT team made clear follow-up plans by contacting cooperating OTPs ahead of time. Patients were excluded from the study if they ended up not discharging as planned and the ED bridge was no longer required, or if patients declined to return. These patients were manually tracked by chart review to determine whether they returned to the ED for dosing over the period from July 2019–July 2022.

The “ED bridge” process consisted of 1) instructing the patient to return to the ED every day starting the morning following the day of discharge for methadone administration until the day of planned OTP intake or return (maximum three days); 2) writing a care plan note in the chart notifying the ED of the dosing plan, days of dosing, and policy; 3) entering an expected arrival notification on the ED track board; and 4) triaging the patient on arrival to a low-acuity part of the ED for methadone dosing and immediate discharge as long as they did not appear to be intoxicated or have another complaint.

A templated note for the “ED bridge” care plan (Appendix 1) was approved by the hospital’s Pharmacy and Therapeutics Committee to provide consistency for the process. It included a dot phrase for a note template that the emergency clinician could also use when the patient returned. The electronic health record (EHR) used in our hospital is Epic (Epic Systems Corporation, Verona WI). Our hospital’s methadone policy was amended to include the ED bridge pathway and approved by our hospital’s compliance and legal offices. The pharmacy department disseminated hospital-wide notification about the policy updates and provided education about the new process to prescribers, pharmacists, nurses, and clinical staff. This study received institutional review board approval.

The primary outcome measurements were the patient return rate to the ED for dosing and the number of doses completed. An ED bridge was considered successful if the patient came for dosing on all planned days; partially successful if they came for dosing on some of the planned days but missed days of dosing; and unsuccessful if they did not come for any of the planned days of dosing. Outcomes and demographic data are expressed by descriptive statistics.

## RESULTS

There were 53 planned ED bridges set up for 47 unique patients. One ED bridge was excluded after the patient stayed through the weekend and didn’t require it. Several patients used the ED bridge workflow more than once due to repeated hospitalizations: three patients used it twice, and one patient used it three times. Demographic characteristics of the 52 planned bridges are summarized in the Table. All the patients with OUD who used this workflow were using heroin.

**Table.** Characteristics of participants in the emergency department bridge program for post-discharge methadone dosing.

Characteristics (at time of ED bridge)	Total (n = 52)	Successful bridge (n = 21)	Partially successful (n = 9)	Unsuccessful (n = 22)
Age				
Average (years)	44.6	47.9	45.1	40.5
Range (years)	29 – 69	29 – 69	31 – 61	29 – 64
Housing status				
Unhoused	25%	28.6%	11.1%	27.3%
Race				
White	48%	28.6%	44.4%	68.2%
Black	42.3%	52.4%	55.6%	27.3%
Hispanic/LatinX	7.7%	19%	0%	0%
Other	1.9%	0%	0%	0%
Gender				
Female	46.2%	38.1%	44.4%	54.5%
Male	58.3%	61.9%	55.6%	45.5%
Route of opioid use				
Stable recovery/ no active drug use	3.8%	4.8%	11.1%	0%
Intranasal only	48.1%	57.1%	44.4%	40.9%
Intravenous	48.1%	38.1%	44.4%	59.1%
Insurance				
Government	98.1%	100%	100%	95.5%
Uninsured	1.9%	0%	0%	4.5%
Methadone program status				
New	76.9%	61.9%	100%	81.8%
Established	23.1%	38.1%	0%	18.2%
Average # of bridge days planned (days)	1.8	1.3	2.8	1.9
Average # of bridge days completed (days)	0.8	1.3	1.3	0

*ED*, emergency department.

Of the 52 planned ED bridges, 21 patients completed all necessary bridge doses (40.4%). Nine patients (17.3%) returned to the ED for at least one day but didn’t present for all planned days. The remaining plans were not successful because 22 patients (42%) either did not return to the ED or left the ED before receiving one dose. In total, 94 visits for methadone dosing in the ED were planned via the ED bridge workflow, and 40 visits actually occurred. The average ED length of stay (LOS) from triage to discharge was 120 minutes, with a range of 36-682 minutes. Six of the 40 visits required full evaluations for additional complaints. Excluding these six visits, the average ED LOS was 89 minutes. Of the 52 planned ED bridges, the average number of days required to complete linkage to treatment was 1.8 days. For patients who successfully completed all necessary bridge doses, the average number of days for linkage was 1.3 days.

Patients were linked to one of 10 methadone clinics, all of which accepted patients with Medicaid. Eight patients who were already established in a methadone clinic accounted for 38.1% of successful linkages.

## DISCUSSION

For the purposes of this study, a patient was defined as a “new” methadone patient if they were not enrolled in a clinic prior to their admission to the hospital and as an “established” patient if they were. The terms “new” and “established” were not descriptors of stability in treatment because occasionally even established patients needed to be newly restarted on methadone due to missing doses at their established OTP, and the outcomes of whether they complied with the ED bridge plan were essentially similar between the two groups. Because our project lacked follow-up with patients at a later timepoint, we were unable to discern the reason for patients not returning to the ED.

“Success” was defined as the patient returning for all planned days. There didn’t appear to be any demographic factor that correlated with the success of the bridge, although this study was not powered to look for any statistical trends. The clearest explanation from the data we were able to collect is that if a bridge plan was shorter, it was more likely to be successful. On average, patients returned for approximately one day. Plans longer than one day were less likely to be successful. Nearly half of the 10 unsuccessful bridge plans occurred within a relatively short four-month time span (September–December 2021). Emergency department wait times and the COVID-19 pandemic may have contributed to this high rate of unsuccessful bridge doses during that time.

Prior to instituting the ED bridge process in our center, we would routinely hold patients committed to treatment in the hospital to ensure linkage to a methadone clinic with no missed doses to decrease the patients’ risk of relapse, overdose, and death upon discharge. The ED bridge process allowed greater flexibility: patients who were committed to treatment but were ready for discharge otherwise could leave and come back for dosing; patients who were getting placed in post-acute care settings but needed to transport for methadone could now transport back to the ED for dosing, thereby allowing weekend discharges; and even patients who were leaving against medical advice were offered the opportunity to dose in the ED to reinforce the message that MOUD is a priority. While it is difficult to determine whether every ED bridge plan decreased LOS, the fact that 40 visits to our ED for methadone dosing did occur via the ED bridge process suggests that we did decrease inpatient hospital days and that this mitigated the increased use of ED resources for these visits.

Instituting the ED bridge workflow was an adjustment for the ED staff. Since there was no pop-up in the EHR, the triage nurses at times needed to be reminded to look for an expected arrival note and to be reminded that these patients could be triaged to the low acuity part of the ED. Most clinicians wrote standard ED notes and did not use the pre-formed templated note for a methadone visit. It took some time for all staff members to get used to the new workflow, which likely explained the average LOS being approximately 1.5 hours when a full evaluation was not required. The LOS also accounted for time spent in the waiting room and clinicians ordering methadone and providing discharge instructions. It was not 1.5 hours of observation after the dose was given. Based on our team’s experiences with teaching the workflow, it appeared that the ED staff was receptive to the overall idea, in part because our institution had gotten used to the culture of the emergency physician-led SUIT team. During the COVID-19 pandemic, there was also turnover in the ED nurse workforce that necessitated re-trainings on the workflow, which could have also contributed to the wide variation in LOS.

This study took place in a large urban environment from 2019–2022, a period that not only encompassed the COVID-19 pandemic but also the continued worsening of the opioid epidemic. During that time, there were significant and evolving changes to how OTPs functioned due to COVID-19 emergency conditions and to the desire to reduce barriers to treatment. The OTPs changed their intake process, sometimes several times throughout that period, at first to be more restrictive^12^ and then later to allow flexibility. Prior to this period, a typical OTP had specific days designated for intake appointments. Intakes could take approximately one hour, and a patient may not have actually started dosing on that day. Patients were often instructed to return a few days later to then meet with the clinician to start their methadone titration.

The typical initial dosing schedule is daily dosing Monday through Saturday with a take-home dose dispensed on Saturday for use on Sunday when the OTP was closed. Initially our SUIT program was able to help patients complete phone intakes while hospitalized; however, this protocol later evolved to match the changes in OTPs, which developed expanded days for walk-in intakes. Several OTPs also changed their workflows regarding day of intake and day of first dose, and sometimes we had to use our ED bridge protocol to keep dosing patients during the gap between the day of their intake and the day of their first dose. During this period, OTPs also permitted more take-home methadone doses, sometimes switching to Monday-Wednesday-Friday dosing schedules with every other day take-home doses, weekly dosing schedules with six days of take-home doses, or even monthly dosing with 27 days of take-home doses. This allowed patients to not have to go to the OTP as often, facilitating social distancing, but it also led to greater access to diverted methadone. The goal of our “ED bridge” workflow was to decrease dose disruption by providing a way for patients to obtain methadone safely while complying with dispensing restrictions. It is possible patients obtained methadone through other means and, thus, did not return for the ED bridge.

One OTP in our urban area decreased the barriers to entry significantly over this time period: they expanded intakes to Monday through Friday; allowed dosing even before full completion of intake; did not require photo ID as long as the patient had identifying paperwork (including hospital discharge papers); and accepted all forms of government insurance. This OTP ended up becoming the default option that we could rely on when setting up our ED bridge plans, even though we still did use the workflow for linking to other OTPs as well. In areas of the country with more limited and restrictive access to methadone OTPs, our three-day ED bridge model may not be as feasible.

## LIMITATIONS

This study took place in an urban area with federal and state support for OTPs. We did not look at patient follow-through for OTP intakes or retention in long-term treatment. Another limitation is that feedback from ED staff on this new workflow was not collected to fully assess attitudes and barriers.

## CONCLUSION

Expanding access to methadone remains an issue for policymakers and advocates. Ideas such as mobile clinics, new guidelines suggesting limited dispensing, and proposals to allow standard commercial pharmacies to dispense methadone are all ongoing considerations.^13^ Our ED bridge workflow, however, allows us to expand access and continuation of methadone using existing laws and regulations, and to better use hospitals as a point of entry into methadone treatment.

## Supplementary Information



