# Supplementary material for: A Novel Use of the “3-Day Rule”: Post-discharge Methadone Dosing in the Emergency Department
Source: West J Emerg Med. 2024 Jun 11;25(4):477–82. doi: 10.5811/westjem.18030 (PMC11254140; doi:10.5811/westjem.18030)
Supplement: Supplementary file 1 [file wjem-25-477-s001.docx]

**APPENDIX**

SUIT Methadone Follow-up Care Plan

***, has received a dose of ***mg methadone for treatment of opioid withdrawal and maintenance while hospitalized.

Patient has a plan for continuation of methadone maintenance at an opioid treatment program on ***.

Patient is expected to require:***mg in the ED on Dates: ***

NO further methadone after:  *** (unless reauthorized by SUIT)

ED team may use dot phrase ".rushedmethadone" as a template for a physician or advanced practice clinician note for methadone administration in the ED.

Please be sure to have the nurse do a mouth check after the methadone pills are swallowed.

ED Social Work Intervention Required: {Yes No}

Please Refer to Inpatient Methadone Ordering Policy OP-0441 as needed regarding this treatment plan.

- Per this policy the patient may receive the dose of methadone immediately in rapid assessment or low resource and be discharged without testing or observation if the patient:

                   -Does not appear intoxicated in the ED

                   -Denies intoxication after last dose of methadone

                   -Has a clear care plan established by SUIT

                   -Has no other complaints requiring further medical screening exam

Please page SUIT with questions
